# Frontal Lobe Neurocysticercosis Presenting With Seizures and Anxiety: A Case Report Highlighting Radiological–Psychological Dimensions

**DOI:** 10.7759/cureus.97015

**Published:** 2025-11-16

**Authors:** Nisha P Govindani, Hrishikesh M Gaikwad

**Affiliations:** 1 Radiology, Mahatma Gandhi Mission (MGM) School of Biomedical Sciences, Chhatrapati Sambhajinagar, IND; 2 Medical Laboratory Technology, Mahatma Gandhi Mission (MGM) School of Biomedical Sciences, Chhatrapati Sambhajinagar, IND

**Keywords:** epilepsy by ncc, magnetic resonance imaging (mri), neurocysticercosis (ncc), psychiatric comorbidities, seizures

## Abstract

Neurocysticercosis (NCC) is a major cause of acquired epilepsy in endemic regions. While seizures are the predominant manifestation, psychological distress is often underrecognized and rarely quantified in single-lesion cases. We describe the case of a 20-year-old male from rural India with a six-month history of recurrent generalized tonic-clonic seizures, accompanied by social withdrawal and anxiety. Neurological examination was unremarkable. Brain MRI demonstrated a solitary right frontal ring-enhancing lesion with perilesional edema, consistent with parenchymal NCC in the colloid vesicular stage. Psychological assessment revealed a Generalized Anxiety Disorder-7 (GAD-7) score of 7, indicating mild anxiety. Although the anxiety was mild by scale, it was functionally significant, leading to avoidance of academic and social activities and reduced self-esteem. This case highlights that even a solitary parenchymal NCC lesion can produce disabling psychological distress alongside seizures. Quantifying anxiety with validated scales underscores the feasibility and clinical value of structured psychological assessment in NCC. Psychological assessment should be mandatory in NCC management, particularly in endemic and resource-limited settings, to optimize outcomes and reduce stigma.

## Introduction

Neurocysticercosis (NCC), caused by infection with the larval stage of *Taenia solium*, is the most common parasitic infection of the central nervous system and the leading cause of acquired epilepsy worldwide [1,2]. Its burden is particularly pronounced in endemic regions such as Latin America, sub-Saharan Africa, and South Asia, where radiological evidence of NCC is observed in up to one-third of individuals with epilepsy, underscoring its major public health significance [3]. Seizures are the hallmark clinical manifestation, reported in 70-90% of symptomatic patients, especially during degenerative stages when inflammation and gliosis enhance cortical excitability [4,5]. Classical reviews have also underscored the evolving knowledge base around NCC, particularly in relation to its neurological and systemic impact [6]. Magnetic resonance imaging (MRI) remains pivotal for diagnosis, allowing precise staging of cysts and distinction from tuberculomas, neoplasms, or other ring-enhancing lesions [7]. Revised diagnostic criteria proposed by Del Brutto et al. have further standardized case definitions and improved diagnostic accuracy [8].

Beyond seizures, NCC is increasingly recognized as a disorder with important psychological and cognitive consequences. Neuropsychological syndromes, including depression, anxiety, psychosis, and cognitive impairment, affect approximately 15-20% of patients overall, with higher prevalence among those with epilepsy [8,9]. In an Indian case-control study, psychological comorbidities were documented in 68% of epilepsy patients with NCC compared to 44% of epilepsy patients without NCC, suggesting a distinct neuropsychological signature linked to the infection [10]. Lesion location also appears contributory; frontal and parietal involvement has been associated with increased risk of anxiety and mood disturbances [11]. Broader reviews of parasitic infections similarly highlight the neuropsychological sequelae of NCC and emphasize its under-recognition in routine clinical practice [12]. A recent systematic review and meta-analysis further confirmed that individuals with epilepsy carry a substantially higher burden of psychological comorbidity than those without epilepsy, reinforcing the bidirectional relationship between seizures and psychological illness [13,14]. Despite such evidence, psychological evaluation is rarely incorporated into routine NCC care, particularly in resource-limited endemic settings, resulting in frequent underdiagnosis and perpetuation of stigma, social withdrawal, and impaired quality of life [15]. More recent European experience has additionally highlighted the ongoing diagnostic challenges posed by NCC in non-endemic regions, emphasizing the need for heightened awareness even outside classical endemic zones [16].

Few published case reports have systematically examined psychological manifestations in NCC using validated psychometric instruments. Most descriptions rely on narrative impressions rather than structured assessments, limiting reproducibility and clinical utility [8,9]. Objective tools can contextualize psychological symptoms, highlight their functional significance, and support early intervention. Among these, the Generalized Anxiety Disorder-7 (GAD-7) scale is a well-validated screening measure for generalized anxiety, with robust psychometric properties and ease of clinical application [17]. Its use in the setting of NCC, however, remains rare, particularly in cases with solitary lesions.

The present report describes the case of a young male with parenchymal NCC presenting with recurrent seizures and functionally disabling anxiety quantified using the GAD-7. By integrating radiological findings with structured psychological evaluation, this case illustrates the heterogeneity of NCC presentations and emphasizes the need for multidisciplinary management strategies that encompass neurological, radiological, and psychological dimensions of care.

## Case presentation

Patient information

A 20-year-old right-handed male from rural central India presented with a six-month history of recurrent generalized tonic-clonic seizures (GTCS). Seizures began abruptly without preceding febrile illness, trauma, or substance use. Each episode lasted 1-2 minutes with 10-15 minutes of post-ictal confusion. They recurred two to three times per month, including two in public, resulting in embarrassment and marked social withdrawal.

Additional symptoms included intermittent headaches, occasional giddiness, transient blurred vision, and nausea. There was no personal or family history of epilepsy or psychological illness. Neurological examination was normal, with intact higher functions, cranial nerves, and motor and sensory systems, and no evidence of papilledema.

Examination/investigation

**Figure 1:**
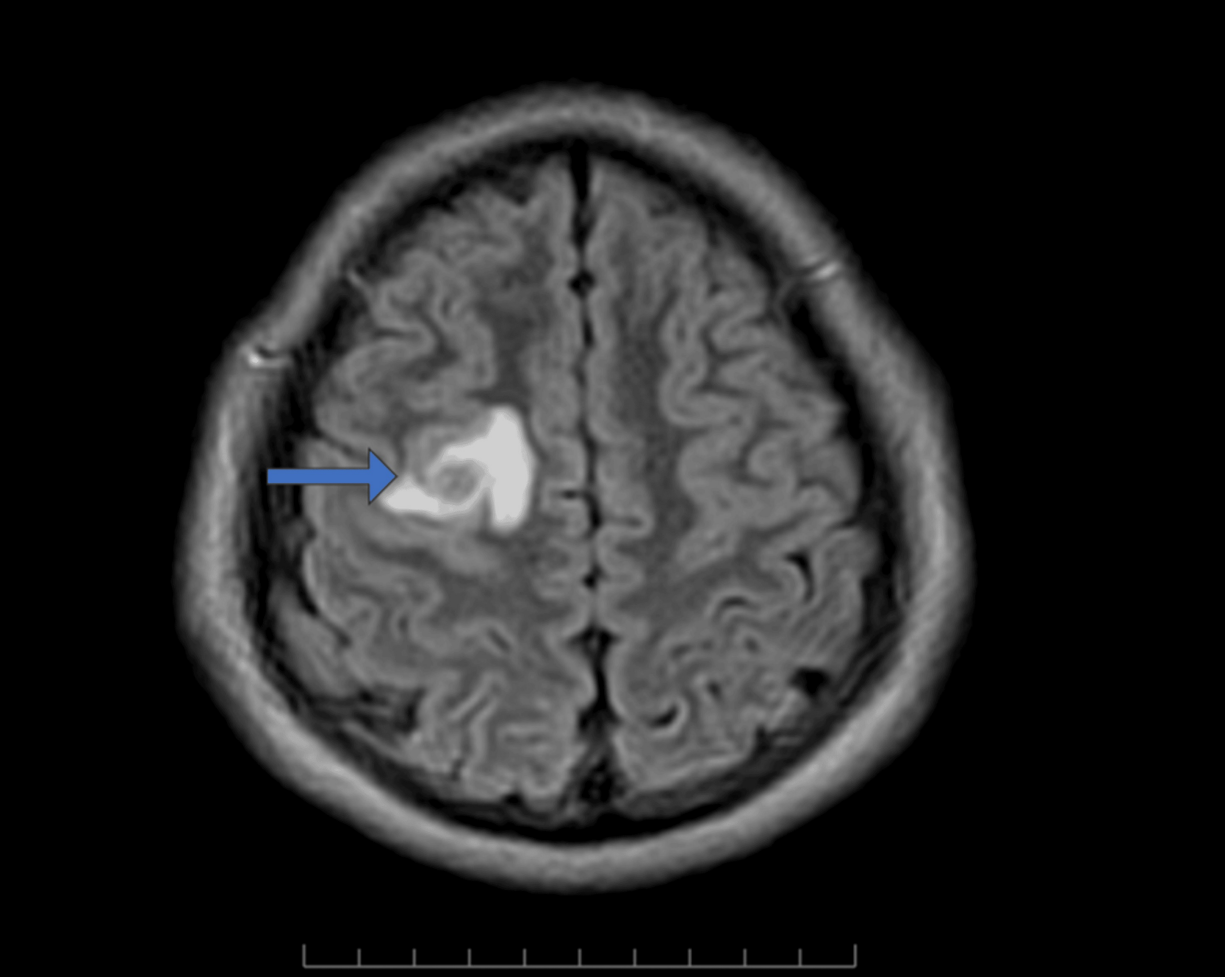
Axial FLAIR MRI image showing a central hypointense lesion with surrounding perilesional hyperintense edema (arrow). FLAIR, fluid-attenuated inversion recovery

**Figure 2:**
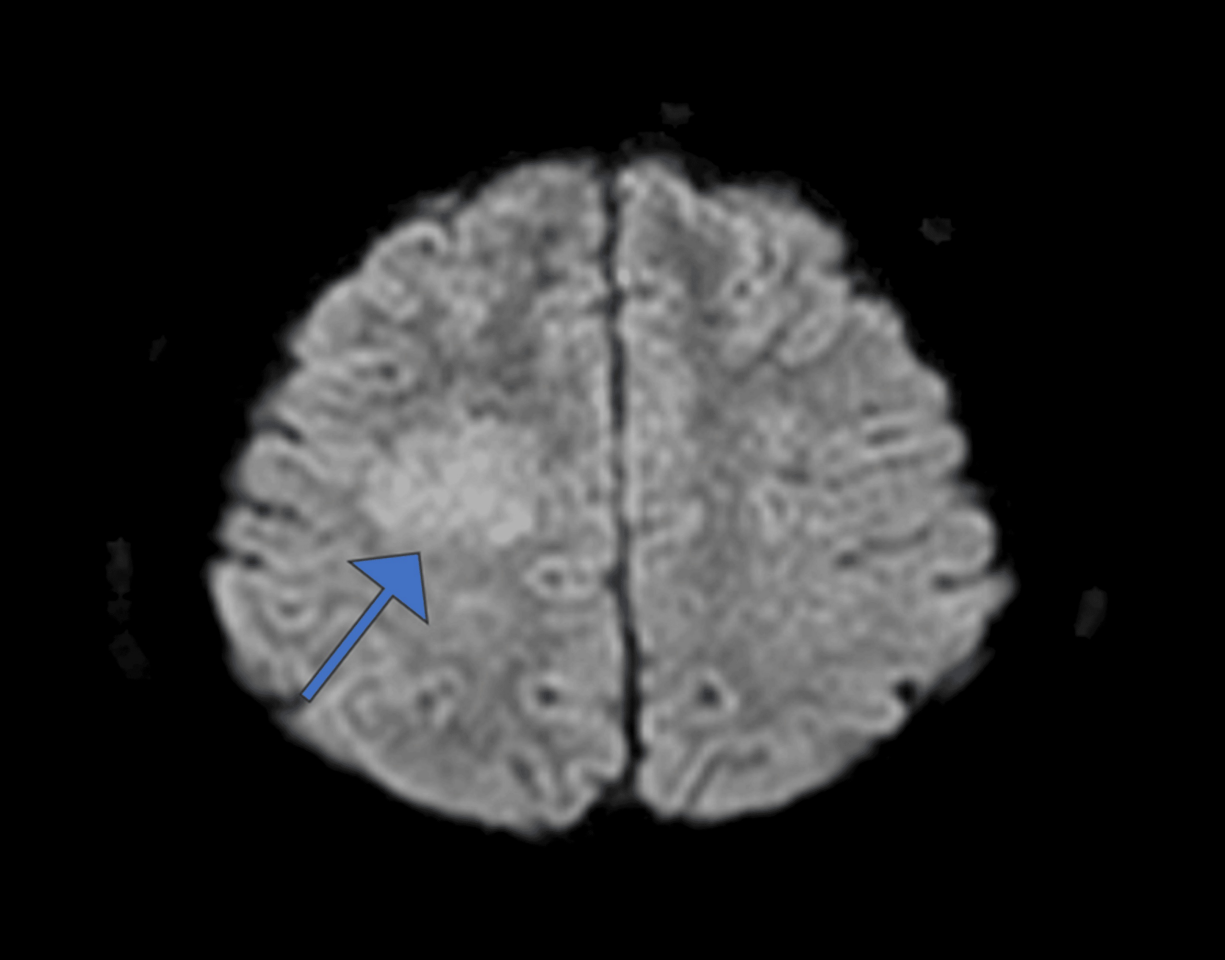
Axial diffusion-weighted MRI image showing no significant diffusion restriction at the lesion site (arrow).

**Figure 3:**
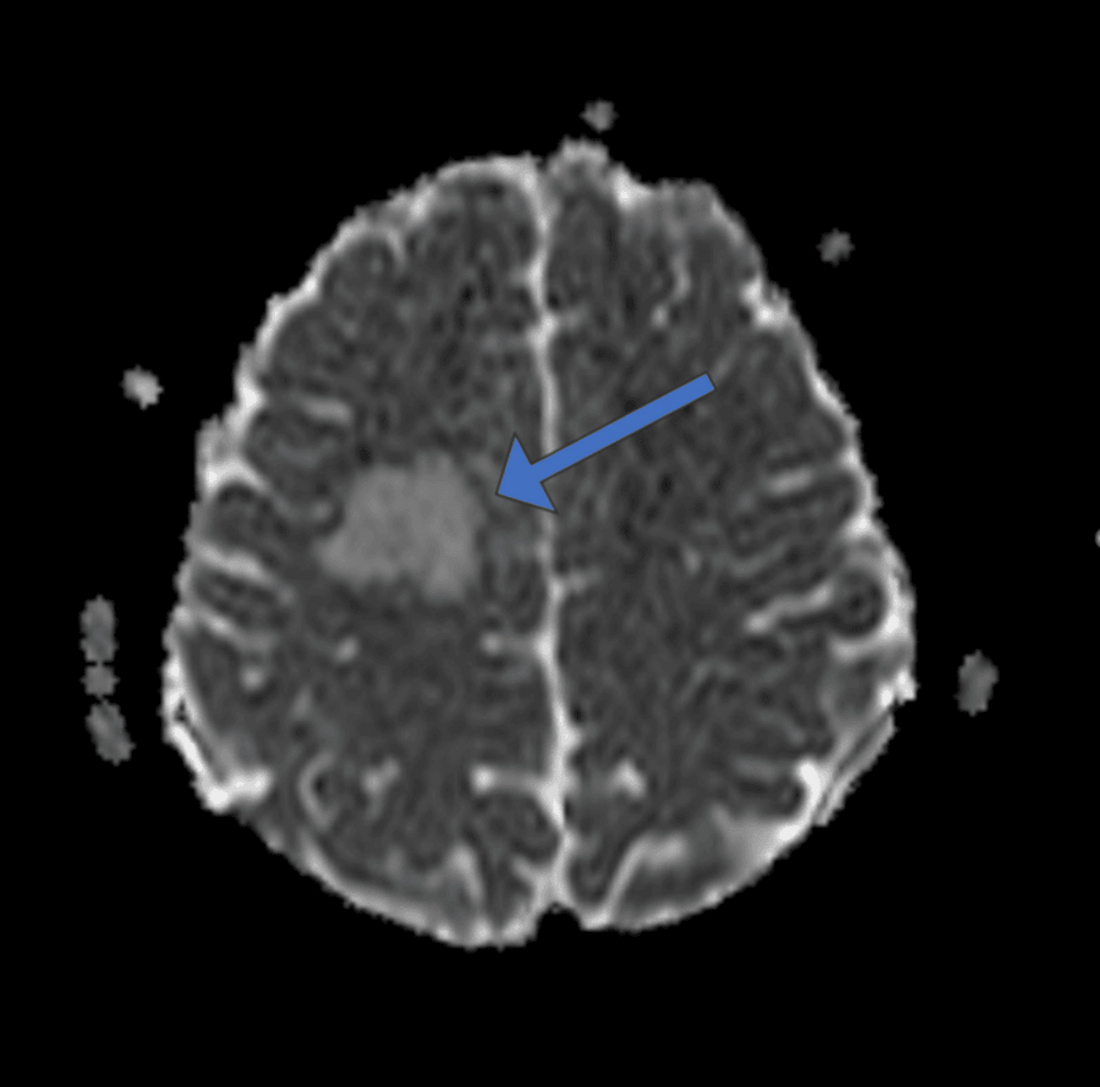
Axial ADC MRI image showing higher ADC values with corresponding hyperintensity at the lesion site in the right high frontal lobe (arrow). ADC, apparent diffusion coefficient

**Figure 4:**
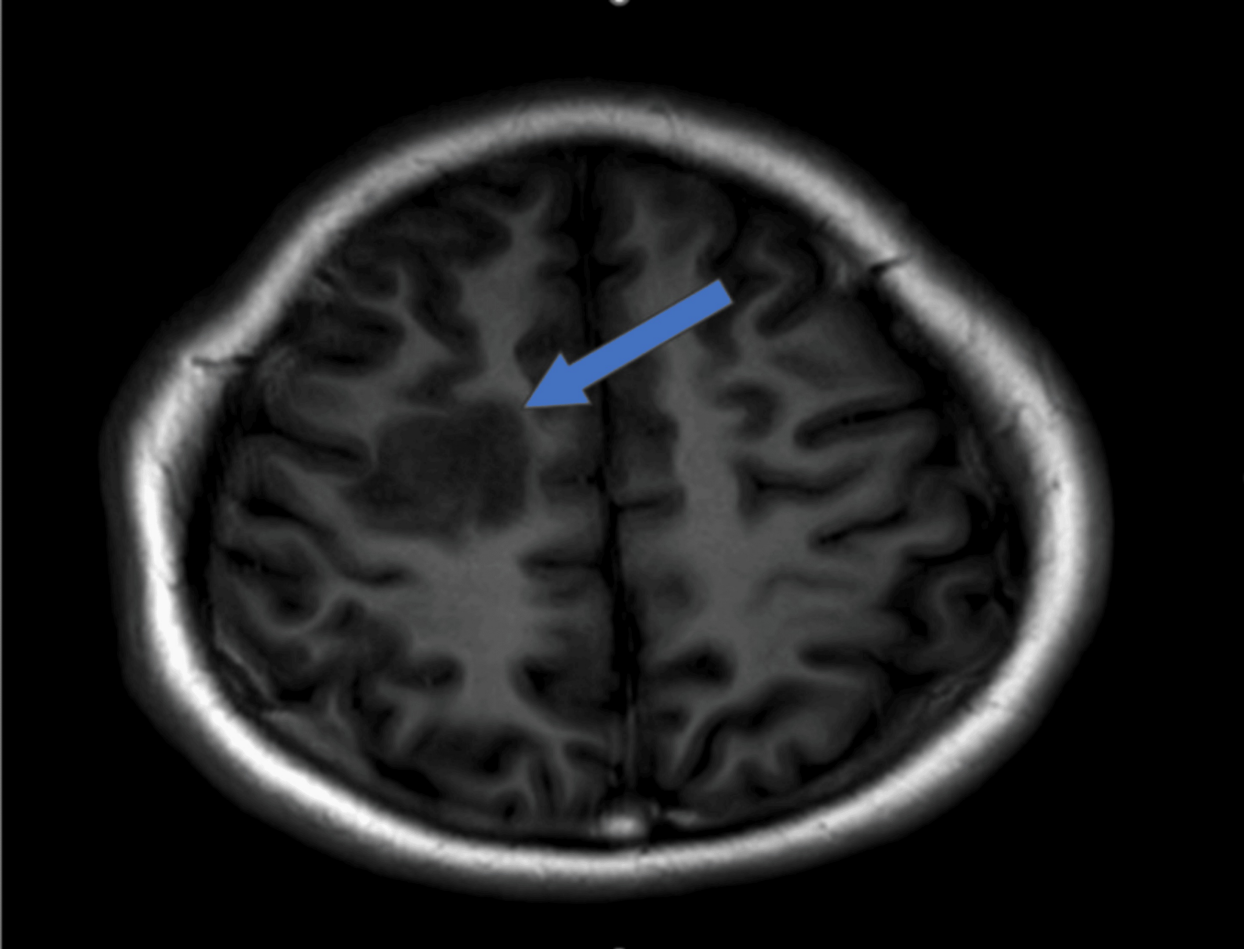
Non-contrast axial T1-weighted MRI image showing a heterogeneous hypointense lesion in the right high frontal lobe at the gray-white matter junction (arrow).

**Figure 5:**
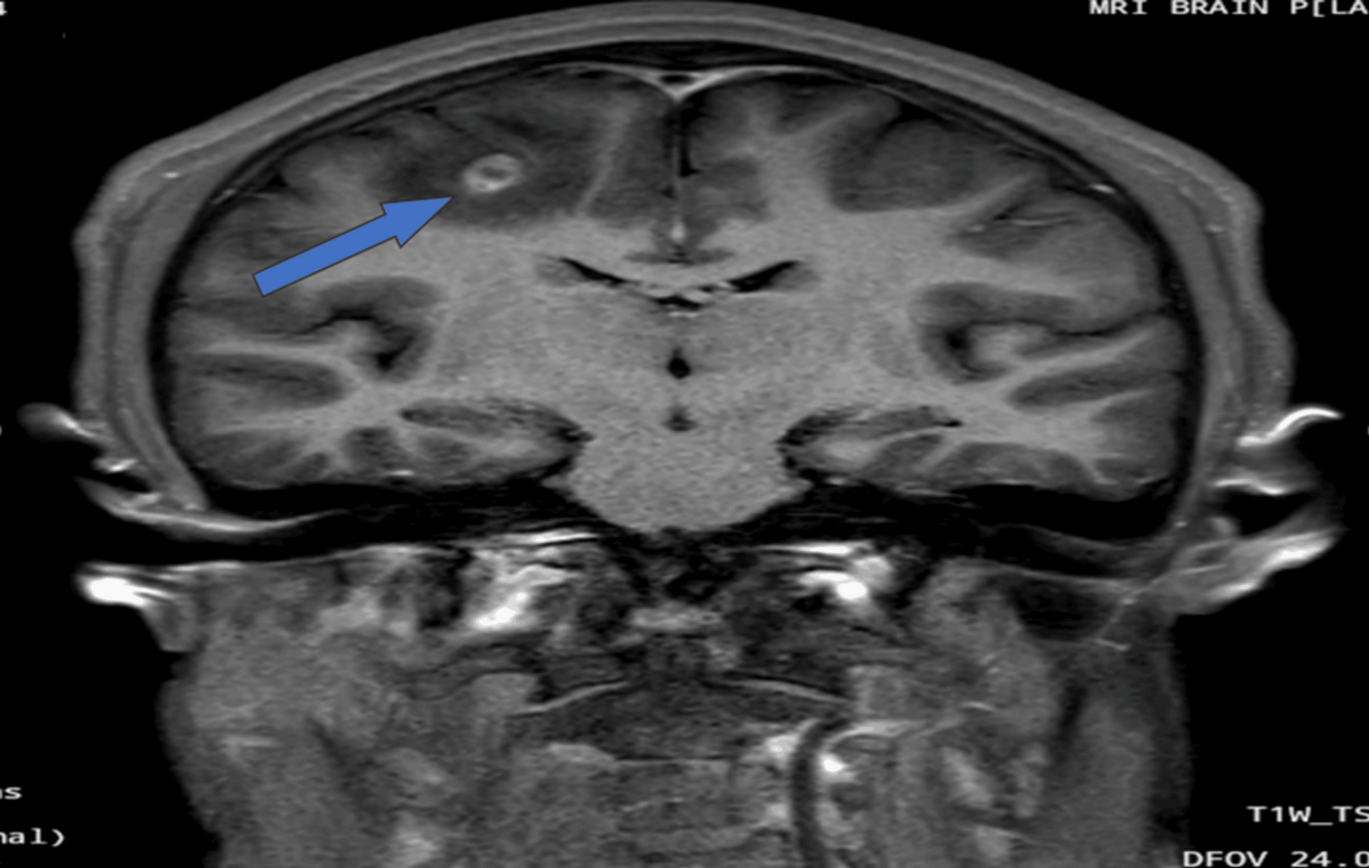
Post-contrast coronal T1-weighted MRI image showing an intensely ring-enhancing lesion in the right high frontal lobe at the gray-white matter junction, with surrounding non-enhancing hypointense perilesional edema (arrow).

Magnetic resonance imaging (3-Tesla, gadolinium contrast) revealed a solitary 7 × 7 × 7.2 mm lesion at the right frontal gray-white junction. The lesion appeared hypointense on T1 and hyperintense with a hypointense rim on T2. It showed thick ring enhancement post-contrast with perilesional edema, without calcification, hemorrhage, or mass effect - findings consistent with the colloid vesicular stage of parenchymal NCC. The described imaging findings are illustrated in Figures 1-5.

Psychological evaluation was prompted by social withdrawal. The GAD-7 score was 7 (mild anxiety). Despite the mild score, the anxiety was functionally disabling: the patient avoided college attendance and social activities due to fear of seizures and reported reduced self-esteem. This discrepancy between scale score and functional impairment highlights the need for contextual interpretation of psychometric tools in NCC. Clinical interview confirmed anticipatory fear but no suicidal ideation or psychotic symptoms. Routine blood tests were within normal limits. Serological testing for *Taenia solium* was unavailable locally.

Based on clinical, radiological, and psychological findings, a final diagnosis of parenchymal NCC in the colloid vesicular stage with secondary epilepsy and comorbid anxiety manifestation was established (Table 1).

**Table 1:** Clinical, radiological, and psychological findings GTCS, generalized tonic–clonic seizures; MRI, magnetic resonance imaging; GAD-7, Generalized Anxiety Disorder-7; NCC, neurocysticercosis

Domain	Findings
Demographics	20-year-old right-handed male, rural central India
Seizure profile	GTCS, 1–2 min, post-ictal 10–15 min, 2–3/month, socially disabling
Associated symptoms	Headache, giddiness, blurred vision, nausea
Examination	Normal neurological exam; no papilledema
Imaging (MRI 3T)	Solitary right frontal lesion (7 × 7 × 7.2 mm), ring-enhancing, perilesional edema; colloid vesicular stage
Psychological assessment	GAD-7: 7 (mild but functionally disabling); anticipatory fear, social avoidance, reduced self-esteem
Investigations	Routine labs normal; serology unavailable
Final diagnosis	Parenchymal NCC (colloid vesicular stage) with secondary epilepsy and comorbid anxiety

Routine laboratory tests were within normal limits. Serological testing for *Taenia solium* was not available locally, limiting biochemical confirmation.

Therapeutic intervention/treatment

The management plan included albendazole at 15 mg/kg/day for a duration of 28 days as the primary antiparasitic therapy. Prednisolone was added at 0.5-1 mg/kg/day and then tapered gradually over four weeks to control inflammation. For seizure prevention, the patient was placed on levetiracetam in a dosage range of 500 to 1,000 mg daily. Alongside medical treatment, weekly sessions of cognitive-behavioral counselling were provided by a clinical psychologist, focusing on reducing anticipatory anxiety, strengthening coping skills, and assisting the patient’s return to academic and social functioning.

Clinical outcome and follow-up

At the two-month follow-up, the patient remained seizure-free and demonstrated clear improvement in anxiety symptoms, with the GAD-7 score decreasing from 7 to 3. He was able to reintegrate into academic and social life, reporting noticeable enhancement in overall well-being and self-confidence. Because *Taenia solium* serological testing was not accessible locally at the time of diagnosis, a repeat MRI has been planned after completing antiparasitic treatment to evaluate lesion resolution and reinforce diagnostic certainty of NCC. Continued follow-up is underway to determine the appropriate duration of antiparasitic and antiepileptic therapy.

## Discussion

This case illustrates how even a solitary parenchymal NCC lesion can precipitate disabling psychological distress when assessed systematically. Seizures remain the most frequent clinical manifestation of NCC, but psychological and cognitive symptoms such as anxiety, depression, psychosis, and cognitive decline are increasingly recognized as significant contributors to overall morbidity [7-9,12]. Despite this, psychological sequelae are often underdiagnosed in endemic, resource-limited regions where clinical attention is predominantly directed toward seizure control [2,11,16].

Magnetic resonance imaging (MRI) is the modality of choice for identifying and staging NCC lesions [4-6,13]. Although MRI is the preferred imaging tool, it is not independently diagnostic, as other ring-enhancing lesions such as tuberculoma or metastasis can appear similar. In our case, a visible scolex (“eccentric dot”) was not observed; however, this feature is not consistently present, particularly in solitary parenchymal lesions. Serological testing for *Taenia solium* was unavailable locally, which limited microbiological confirmation. Therefore, the diagnosis was based on the combination of clinical presentation (new-onset seizures), characteristic MRI findings (solitary right frontal ring-enhancing lesion with perilesional edema), and epidemiologic background, in accordance with the revised Del Brutto criteria for NCC. A follow-up MRI after completion of antiparasitic therapy has been scheduled to document radiological regression, which will serve as supportive diagnostic evidence. In our patient, imaging revealed a colloid vesicular lesion with ring enhancement and perilesional edema - findings typical of the degenerative stage, which is strongly associated with epileptogenic activity [7-9]. The lesion’s right frontal localization provided a plausible neurobiological substrate for psychological symptoms, as disruption of frontal-prefrontal circuits, particularly orbitofrontal-amygdala pathways, has been linked to anticipatory anxiety and fear dysregulation in primary anxiety disorders [10,12]. Thus, neuroimaging not only explained the seizure mechanism but also contextualized the psychological presentation.

Therapeutically, albendazole in combination with corticosteroids remains the cornerstone of management for active parenchymal lesions, aiming to eradicate the parasite and mitigate inflammatory edema [13,14]. Antiepileptic drugs provide symptomatic seizure control, and levetiracetam was effective in this case. Seizure freedom at two months suggests successful suppression of inflammatory hyperexcitability; however, recurrence remains common even after apparent radiological resolution [15]. This underscores the necessity of long-term neurological and radiological follow-up to monitor outcomes.

Psychological distress in NCC has been documented in up to half of patients, yet systematic quantification using validated psychometric instruments is rarely performed [11,16]. Most published reports rely on descriptive accounts, limiting clinical comparability. By employing the GAD-7 scale, we objectively demonstrated that even a “mild” numerical score can represent functionally disabling anxiety in the NCC context [17]. The patient’s avoidance of college and social interactions highlights the importance of interpreting psychometric scores within sociocultural and functional contexts rather than in isolation.

Counselling and psychological support were integral to management. Weekly cognitive-behavioral counselling addressed anticipatory fear, enhanced coping strategies, and facilitated social reintegration. The patient’s reduction in GAD-7 score and return to academic activities reinforce the value of integrating psychological care into NCC management. Without such support, psychological distress may persist despite seizure control, perpetuating stigma and disability [2,11,16].

Optimal management of NCC thus requires a multidisciplinary approach. Radiologists contribute to lesion staging and monitoring, neurologists direct seizure management, and clinical psychologists or mental-health professionals address comorbid psychological manifestations. Embedding psychological assessment within routine NCC care could improve detection, reduce stigma, and enhance quality of life in affected patients. Future research should prioritize two areas: first, prospective studies to clarify whether lesion location predicts psychological outcomes; and, second, longitudinal follow-up to determine whether psychological remission persists once seizures stabilize and lesions resolve radiologically. Such evidence will inform guidelines that extend beyond seizure control to encompass holistic, patient-centered care.

## Conclusions

This case demonstrates that even a solitary parenchymal NCC lesion can produce disabling psychological distress in addition to seizures. We highlight the need for structured mental health evaluation in NCC by quantifying anxiety with validated scales. Psychological assessment must be embedded in NCC management, particularly in endemic, resource-limited settings, where underrecognition perpetuates disability and stigma. Multidisciplinary care that integrates neuroimaging, antiparasitic therapy, seizure control, and psychological support is essential to improve long-term outcomes and quality of life.
